# Optimization of Parameters for a More Efficient Use of Adipose-Derived Stem Cells in Regenerative Medicine Therapies

**DOI:** 10.1155/2012/303610

**Published:** 2012-03-14

**Authors:** Meire Aguena, Roberto Dalto Fanganiello, Luiz Alexandre Lorico Tissiani, Felipe Augusto André Ishiy, Rodrigo Atique, Nivaldo Alonso, Maria Rita Passos-Bueno

**Affiliations:** ^1^Institute of Biosciences, University of São Paulo, 05508-090 São Paulo, Brazil; ^2^Department of Plastic Surgery, Faculty of Medicine, University of São Paulo, 01246903 São Paulo, Brazil

## Abstract

Adipose tissue-derived stem cells (ASCs) association to fat in autologous lipotransfer is promising for a more effective soft tissue reconstruction, and optimization of protocols to isolate ASCs from lipoaspirate fat is much needed. We demonstrated that an increase in adipocyte differentiation is dependent on the number of ASCs. In a sample of 10 donors, we found a higher concentration of nucleated cells in the lower abdomen compared to flank (*P* = 0.015). In a sample of 6 donors we did not find differences in the cell yield obtained by manual or pump-assisted aspiration (*P* = 0.56). We suggest that the increase in the number of ASCs in the reinjected fat may enhance the efficiency of newly formed adipose tissue and that the anatomical region from which to harvest fat tissue needs to be considered to optimize the number of ASCs in the harvested tissue. Finally, pump-assisted aspiration can be used without any significant harm to the viability of cells.

## 1. Introduction

Fat transfer has been used over the past decades as an autologous dynamic filler in plastic surgery rehabilitation in several circumstances, such as in reconstruction of damaged adipose tissue due to burn injury, for craniofacial reconstruction due to congenital defects or trauma, in cancer or other tumors as well as for aesthetic reasons. Although this approach is considered successful, the need of several surgical interventions to reach the aimed result is not unusual. Therefore, the development of alternative protocols to achieve more effective reconstruction of soft tissues is of major interest. Nowadays, a current promise is to enhance adipose tissue survivability by the combination of fat transplantation and stem cell therapy, particularly with the use of adipose tissue-derived stem cells (ASCs), since stem cells potentially ameliorate neovascularization and partially halt inflammatory response [1, 2].

Adipose tissue-derived stem cells (ASCs) can be easily isolated from the stromal-vascular fraction (SVF) of human adipose tissue by simple surgical procedure, can be obtained repeatedly, in large quantities and, in some cases, under local anesthesia, and are capable to undergo *in vitro* differentiation towards osteogenic, adipogenic, neurogenic, myogenic, and chondrogenic lineages when treated with specific factors [3, 4]. Multipotentiality of ASCs makes them interesting and promising candidates for mesodermal defect repair and disease management [5].

Regardless of using fat tissue as a sole fulfillment material or implemented with ASCs, there are several questions yet to be tackled in order to enhance its viability in tissue regeneration therapies. For instance, it is not well understood whether the percentage of ASCs in the reinjected fat tissue interferes in the efficiency of adipose tissue neogenesis. Moreover, the influence of the anatomical donor site in the number and type of mesenchymal cells is still uncertain: while some authors suggest that the subabdominal region is the most enriched site for mesenchymal stem cells (MSCs) [6, 7], others point out the hip as the best tissue to extract MSCs. Another important issue to be evaluated is the harvesting methodology, as some authors suggest that the use of pump-assisted technologies decreases the viability of nucleated cells [8]. Considering that these factors might be controlled by the surgeon, their understanding will certainly have an impact in the final concentration of ASCs contained in the lipoaspirate [9].

In view of the questions listed above, the scopes of this work are to investigate if the proportion of ASCs influences the efficiency of the *in vitro* adipogenesis and to compare the quantity of nucleated cells in the SVF depending on the harvest area and finally, the harvesting methodology.

## 2. Materials and Methods

### 2.1. Ethics Statement

This study was approved by the Institute of Biosciences' Human Research Ethics Committee (permit number 095/2009-FR251136).

### 2.2. Lipoaspiration Surgeries

Lipoaspiration was done by a 10 mL syringe coupled to a cannula with a diameter of 2,5 mm, using the Coleman method [10] or by pump-assisted liposuction regulated at −350 mmHg using a cannula with a diameter of 3 mm. For pump versus manual lipoaspiration comparison experiments, 10 donors were submitted to sub-abdominal liposuction of right side with manual method and the sub-abdominal left side with pump method. For flank versus abdomen comparison experiments, 6 donors were submitted to liposuction in these distinct anatomical sites, with traditional pump suction lipoaspiration method. In order to minimize the differences between individuals, samples taken from the different locations and obtained using the different methods tested were paired in each series of subjects.

### 2.3. Adipose-Derived Stem Cells (ASCs) Isolation and Expansion

Adipose tissue from sub-abdominal and flank subcutaneous lipoaspirates were obtained by traditional pump suction from six healthy women undergoing cosmetic surgery procedures. In another instance, adipose tissue from sub-abdominal region of ten healthy women was isolated by either pump-assisted liposuction, with controlled negative pressure, or manual lipoaspiration. The adipose tissue were washed extensively with sterile phosphate-buffered saline (137 mM NaCl, 2.7 mM KCl, 10 mM NA2HPO4, 2 mM KH2PO4 pH 7.4, reagents from Sigma-Aldrich) to remove contaminating debris and red blood cells. Washed adipose tissue was treated with 0.075% collagenase (type IA; Sigma-Aldrich) in PBS for 40 min at 37°C with gentle agitation) as described previously [4]. The collagenase was inactivated with 2 volumes of HBSS (Invitrogen, CA, USA) and the infranatant was centrifuged for 5 min at 3000 g. Concerning the samples used to test whether there is an influence of harvesting pressure on cell concentration, the cellular pellet for seven samples obtained by pump-assisted liposuction and seven matched samples obtained by manual lipoaspiration were resuspended in DMEM F12 (Invitrogen, CA, USA) supplemented with 15% FBS (Hyclone), 1% non essential aminoacids (Invitrogen, CA, USA), 1% penicillin-streptomicyn (Invitrogen, CA, USA) and seeded on conventional tissue culture flasks for immunophenotypical characterization.

### 2.4. Cell Count

Viable cells were counted using the trypan blue dye exclusion assay. A freshly prepared solution of 10uL trypan blue at 0.05% (Sigma Aldrich) in distilled water was mixed with 10uL of cellular suspension for 5 min., viable cells were counted in a Neubauer chamber using a light microscope (Nikon Eclipse TS100).

### 2.5. Immunophenotyping

Immunophenotype characterization of cell populations was done by flow cytometric analysis. For samples used in the comparison of cell yields depending on the anatomical region, we performed this characterization using the freshly isolated stromal-vascular fraction and each sample analyzed is a pool of the stromal-vascular fraction from five women donors. For samples used in the comparison of cell yields depending on the liposuction technique, the plated cell cultures were washed with PBS and digested by trypsin solution (0,125% trypsin, 0,02%EDTA in PBS). The cells were incubated for 1 hour at 4°C with the following anti-human antibodies: CD29-PECy5, CD34PerCP, CD31-PE, CD45-FITC, CD90-R-PE, CD73-PE, CD105 (Becton, Dickinson and Company, NJ, EUA) and SH2, SH3, SH4 gently donated by professor Arnold Caplan (Case Western Reserve University). Matched control samples were incubated with PBS only. After a second wash with PBS, samples incubated with non-conjugated primary antibodies were incubated with anti-mouse-PE secondary antibody (Guava Technologies) for additional 15 min at 4°C. Cell suspensions were washed with PBS, fixed with 1% p-formaldehyde (Sigma-Aldrich) and 5.000 labeled cells were analyzed using a Guava EasyCyte flow cytometer running the Guava Express Plus software (Guava Technologies Hayward, CA, USA).

### 2.6. Preparation of Heterogeneous ASC and Mature Fibroblast Culture

A coronal suture periosteal fibroblast cell lineage and an adipose derived stem cell lineage were grown to 80–90% confluence in independent flasks. The cell lineages were treated with 0,125% trypsin, 0,02% EDTA (Invitrogen, CA, USA) and the suspended cells were mixed in different concentrations of these two cell lineages, as following: 5% ASC and 95% fibroblast; 25% ASC and 75% fibroblast; 50% ASC and 50% fibroblast; 75% ASC and 25% fibroblast and 100% ASC only. Cells were plated in a concentration of 10^4^/cm^2^ for each experiment. These mixed cell lineages were seeded in 12 well plates and after 24 hour, the mixed cells were induced to adipogenic differentiation.

### 2.7. *In Vitro* Adipogenic Differentiation

To induce adipocyte differentiation, cells were cultured in adipogenic induction medium DMEM high glucose supplemented with 10% FBS (Gibco-Invitrogen, CA, USA), 1% penicillin-streptomycin (Invitrogen, CA, USA), 1 *μ*M dexamethasone (Sigma-Aldrich), 100 *μ*M indomethacin (Sigma-Aldrich), 500 *μ*M 3-isobutyl-1-methylxanthine (IBMX), and 10 *μ*g/mL insulin (Sigma-Aldrich) for 14 days.

### 2.8. Oil Red O Staining and Quantification

For Oil Red O staining, cells were cultured in 12-well culture plates and, for each experiment; four wells per condition were used. After adipogenic differentiation for 14 days, cells were washed with PBS, fixed in 4% formaldehyde for 1 hour, and then cells were washed with deionized water. After, the cells were washed with 60% isopropanol and the cell plate was dried at room temperature. The cells were stained with 0.6% (w/v) Oil Red O solution (60% isopropanol, 40% water) for 1 h at room temperature. Cells were then washed with deionized water three times to remove unbound dye and photographed. Stained Oil Red O was also eluted with 100% isopropanol (v/v) and quantified by measuring the optical absorbance at 500 nm. Oil Red O staining of undifferentiated cells grown in parallel culture served as the blank sample for this assay.

### 2.9. Statistical Analysis

Continuous variables were either expressed individually or by mean and standard deviation and we used the nonparametric Wilcoxon signed-rank test for paired data in order to assess if the population mean rank differ. To assess the statistical significance of the correlation between pump-assisted and manual liposuction and between the percentage of ASCs in mixed cellular populations and adipogenic differentiation we used two-tailed Pearson's correlation test. Tests with *P* values < 0.05 were considered to be statistically significant. All the applied tests were done by the use of the GraphPad Prism 5 program.

## 3. Results

### 3.1. Influence of the Concentration of Adipose-Derived Stem Cells on the *In Vitro* Adipogenic Differentiation

**Figure 1:**
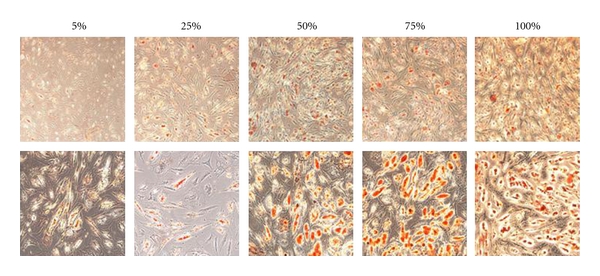
Oil Red-O staining for *in vitro* adipogenic differentiation of the mixed populations of ASCs and mature fibroblasts. Percentages indicate the proportion of ASCs. Pictures taken with 10X of magnification are showed in the upper line and pictures taken with 20X of magnification are showed in the lower line.

**Figure 2:**
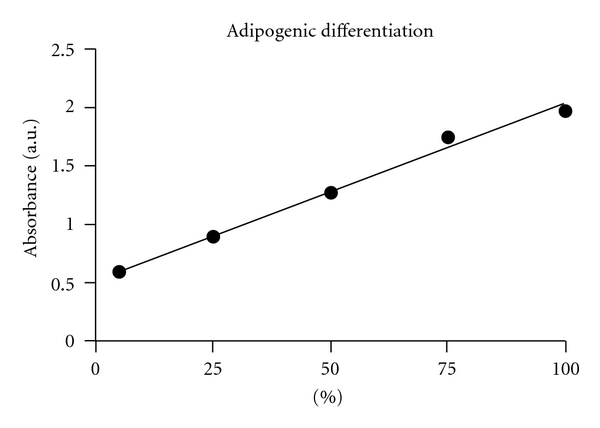
Linear regression showing significant correlation between the percentage of ASCs in the mixed cell population and the increase in Oil Red-O staining (*R* ^2^ = 0.979). Error bars represent standard deviation for quadruplicates of each sample.

To evaluate if there is an influence of the concentration of adipose-derived stem cells on the efficiency of the *in vitro* adipogenesis, we created five heterogeneous cell cultures composed of mixed subpopulations of ASCs and mature fibroblasts derived from cranial coronal suture, induced them to adipogenic differentiation and quantified the lipid vacuoles formation by Oil Red-O staining after 14 days (Figure 1). Applying a linear regression model, as showed in Figure 2, there is a significant correlation between the percentage of ASCs and the increase in Oil Red-O staining (*R* ^2^ = 0.979).

### 3.2. Influence of Donor Site on Cell Concentration

**Figure 3:**
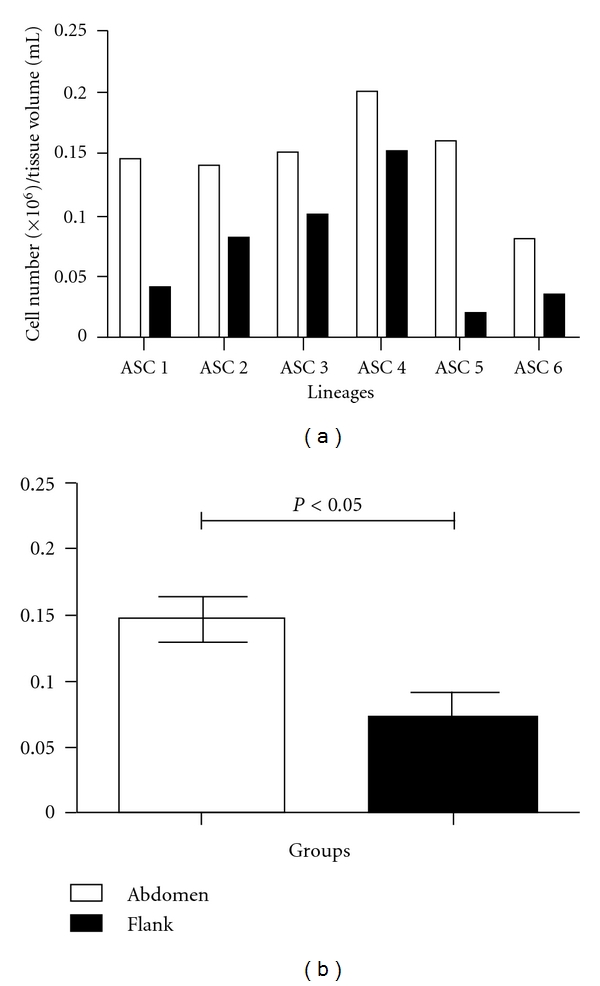
Bar graphs showing (a) cell quantity (×10^6^) per volume of fat tissue (mL) obtained from abdomen or flank from six individuals and (b) Mean and standard deviation from data showed in graph “a”. The Wilcoxon signed-rank test indicates significant difference between the samples (*P* < 0.05).

 In order to determine if the number and proportion of mesenchymal cells varies according to the donor site of the adipose tissue, we evaluated cells from SVF from six female patients with mean age of 37 years (range, 26–51 years) submitted to liposuction at the lower abdomen and at the flank following medical recommendations. We observed a significantly higher concentration of nucleated cells in fat from the lower abdomen when compared to fat from the flank (*P* = 0.015; Figures 3(a) and 3(b)).

**Figure 4:**
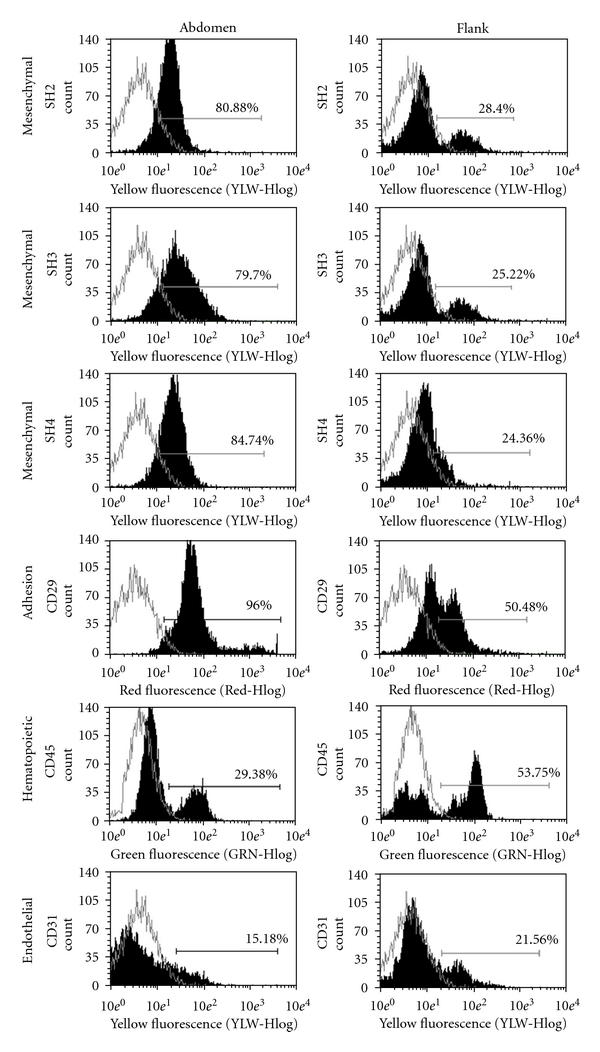
Immunophenotype characterization of fresh human stromal vascular fraction isolated from adipose tissue. The donors were submitted to liposuction in 2 different sites (abdomen and flank), and each sample analyzed is a pool of the stromal-vascular fraction from five women donors. Histogram for each sample (areas displayed in black) comparing cell number and fluorescence intensity with matched negative controls (areas displayed in grey). The percentage of positively stained cells is showed above each histogram.

As showed in Figure 4, we observed a higher proportion of cells positive for mesenchymal (79.7–84.7%) and adhesion cell markers (CD29; 96%) in samples isolated from the abdomen as compared to the samples obtained from the flank (24.36–28.40% and 50.48%, resp.). Furthermore, samples from the flank had a more prominent subpopulation of cells of hematopoietic (53.75%) and endothelial (21.56%) origin when compared to the ones from the abdomen (29.38 and 15.8%, resp.).

### 3.3. Influence of Harvesting Methodology on Cell Concentration after 1 Passage

**Figure 5:**
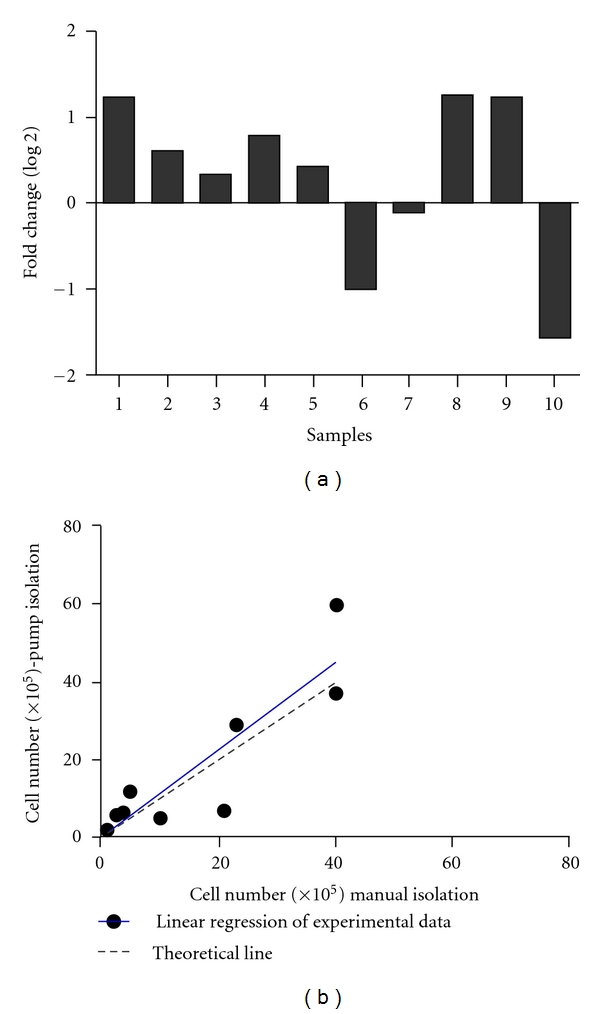
(a) bar graph depicting the log_2_ of the fold changes between the quantity of cells obtained by pump-assisted method compared to the amount of cells obtained by manual aspiration for ten individuals. (b) linear regression model showing no statistical difference in cell number depending on the liposuction method used. The difference in the slopes of the linear regression of experimental data (black line) is not significant (*P* = 0.56) compared to the theoretical line (dotted line, constructed assuming there is no difference between the data).

A group formed by 10 patients, with mean age of 49 (range, 22–73 years) and mean body mass index of 24.88 (range, 22–29.6), submitted to sub-abdominal liposuction according to medical recommendation, was used to evaluate if there is an influence of the harvesting method on the yield of nucleated cells in the SVF. Liposuction was performed applying 2 commonly used harvesting methods: manual aspiration using a syringe and pump-assisted aspiration, with a controlled pressure of −350 mmHg. After 1 passage in culture, cells aspirated manually and obtained by pump-assisted aspiration showed positive staining (>89%) for mesenchymal (SH2, 3 and 4) and adhesion (CD29) markers and negative staining (<4%) for hematopoietic (CD45) and endothelial (CD31) markers. Fold change differences in the amount of cells obtained by pump-assisted method were calculated compared to manual aspiration and are represented in Figure 5(a). Applying a linear regression model (Figure 5(b)), there is no statistically significant difference on the cell yield obtained using these 2 methods.

## 4. Discussion

In autologous fat transplantation with large volume transfer, fat survival rather than fibrosis is desired. Other expected characteristics, which are usually highly variable, are the clinical longevity of fat graft and the maintenance of volume of the transplanted fat. To amend these variables, the combination of stem cell therapy with fat transfer, supplementing fat grafts with adipose-derived stem cells, has been reported as a method of autologous tissue transfer termed cell-assisted lipotransfer [9, 11, 12] and is a promise for the rehabilitation of several patients.

Using an *in vitro* model of admixtured heterogeneous cell populations we found a positive correlation between the percentage of ASCs and the increase in the *in vitro *adipocyte differentiation. Thus, we suggest that the increase in the number of ASCs in the reinjected fat tissue may enhance the efficiency of newly formed adipose tissue. These results would thus support the idea that enrichment of adipose tissue with mesenchymal stem cells can influence the rehabilitation process of patients submitted to autologous fat graft [5].

Therefore, establishing the ideal parameters to optimize the number of viable mesenchymal cells, such as settling preferred donor sites of lipoaspiration from which to isolate the ASCs and determining if the method of lipoaspiration interferes in the quantity and quality of the mesenchymal cells will certainly contribute to a more successful use of fat transplantation enriched with mesenchymal cells.

 The finding of a significantly higher concentration (*P* < 0.05) of nucleated cells in the lower abdomen when compared to flank together with the observation of an enriched subpopulation of cells from mesenchymal origin in samples from the lower abdomen suggests that SVF from the lower abdomen might be a better source of mesenchymal stem cells than adipose tissue isolated from the flank. Our findings are consistent with the work of Padoin et al. [7], where they found a significantly higher concentration of nucleated cells in the SVF obtained from the lower abdomen when compared to upper abdomen, inner thigh, trochanteric region, knee, and flank. Jurgens et al. [6] also suggested, after CFU assays, that the abdomen is preferable to the hip/thigh region for harvesting mesenchymal stem cells.

If we take into account the number and type of mesenchymal cells, it thus seems that the adipose tissue from the lower abdomen is better than those from the flank. However, further studies are necessary in order to elucidate the functional effect on tissue regeneration as we do not know if the higher proportion of hematopoietic and endothelial cells contained in the flank might actually facilitate neovascularization, which is critical for the success of the surgery procedure.

We did not find significant differences in SVF cell yields comparing manual liposuction using a syringe or pump-assisted lipoaspiration. Indeed, Fraser et al. [13] also tested these two approaches of fat tissue aspiration and concluded that the frequency of clonogenic cells was not impacted by the harvesting methodology. Although these data suggest no influence of these methodologies in the SVF nucleated cell quantity, a study by Mojallal et al. [8] concluded that liposuction at a controlled pressure of −350 mmHg gives a greater cell yield when compared to power-assisted aspiration with a negative pressure of 700 mmHg and syringe aspiration. However, these results should be considered with caution, as they performed this test using only 3 patients.

In this paper, we demonstrate that even though there is no difference on the nucleated cell yield obtained by manual aspiration using a syringe or with pump-assisted aspiration with a pressure of −350 mmHg, the anatomical region from which to harvest fat tissue needs to be considered as a means to optimize the total number of nucleated cells in the SVF and, consequently, the quantity of adipose-derived stem and progenitor cells. For the foreseeable future, implementations to the cell-assisted lipotransfer will lead to improve fat-grafting outcomes for restoration of tissues for either aesthetic or reconstructive purposes. Further, our results support the hypothesis that enrichment of adipose tissue with mesenchymal stem cells might improve the regeneration process following a cell-assisted lipotransfer.
